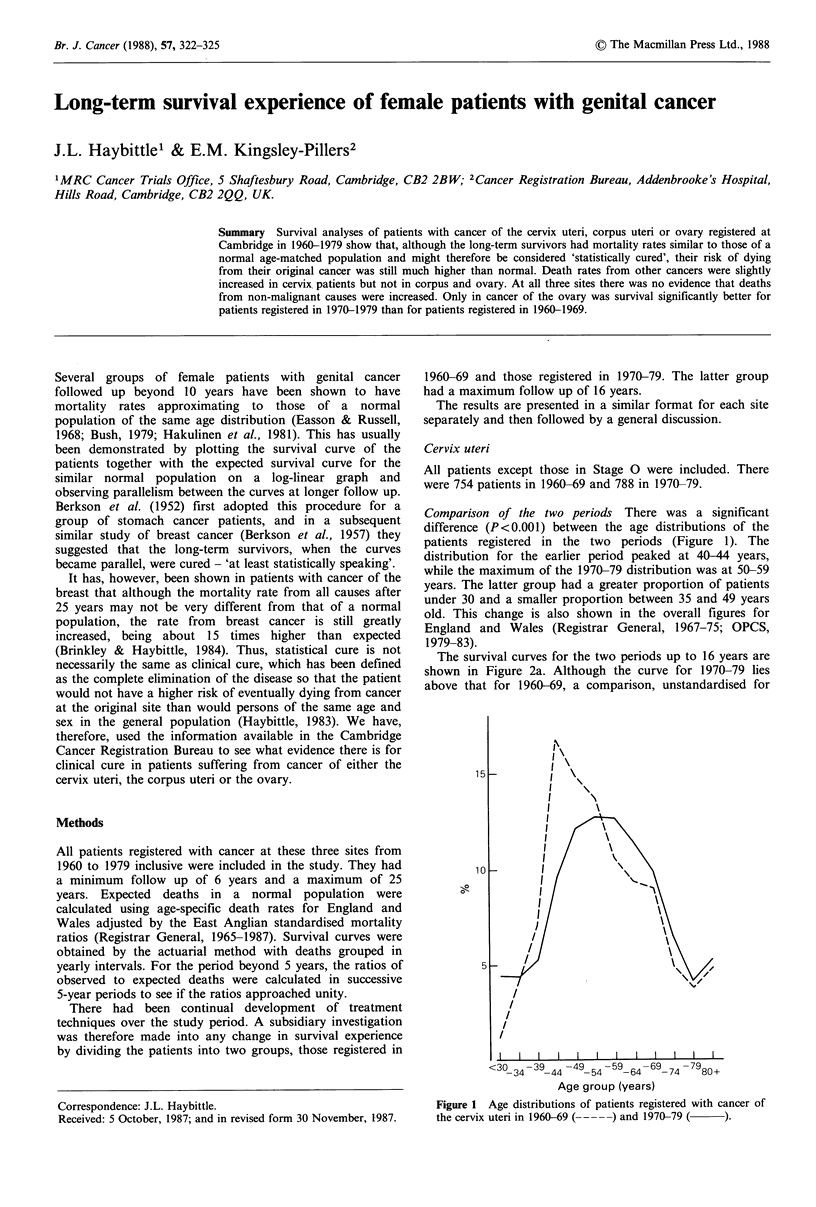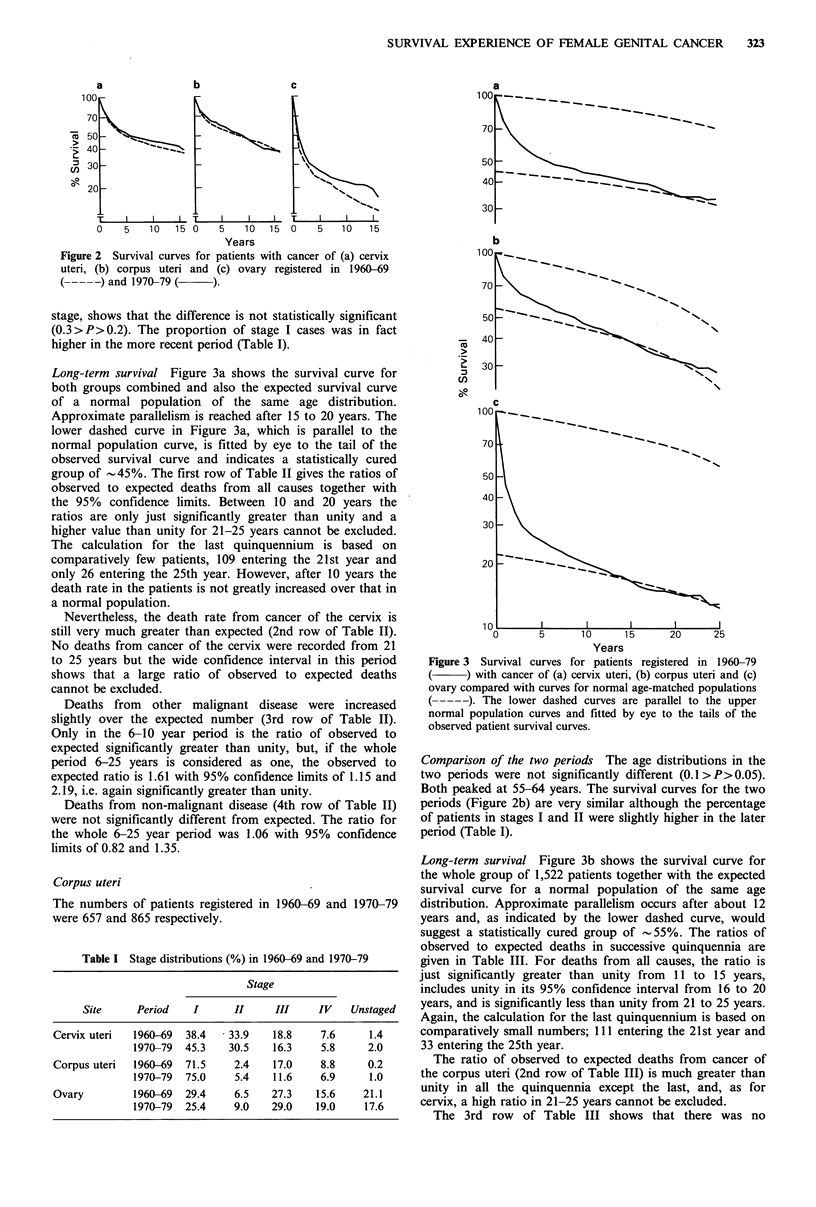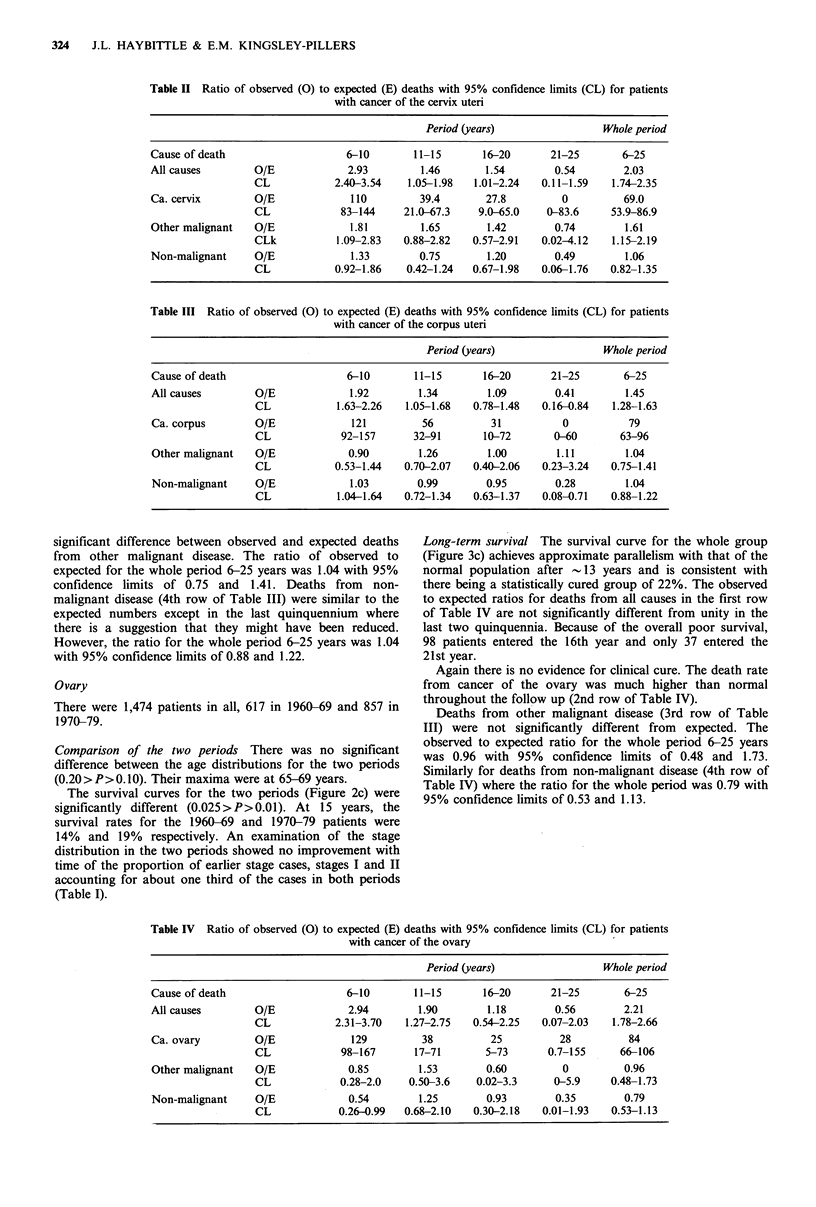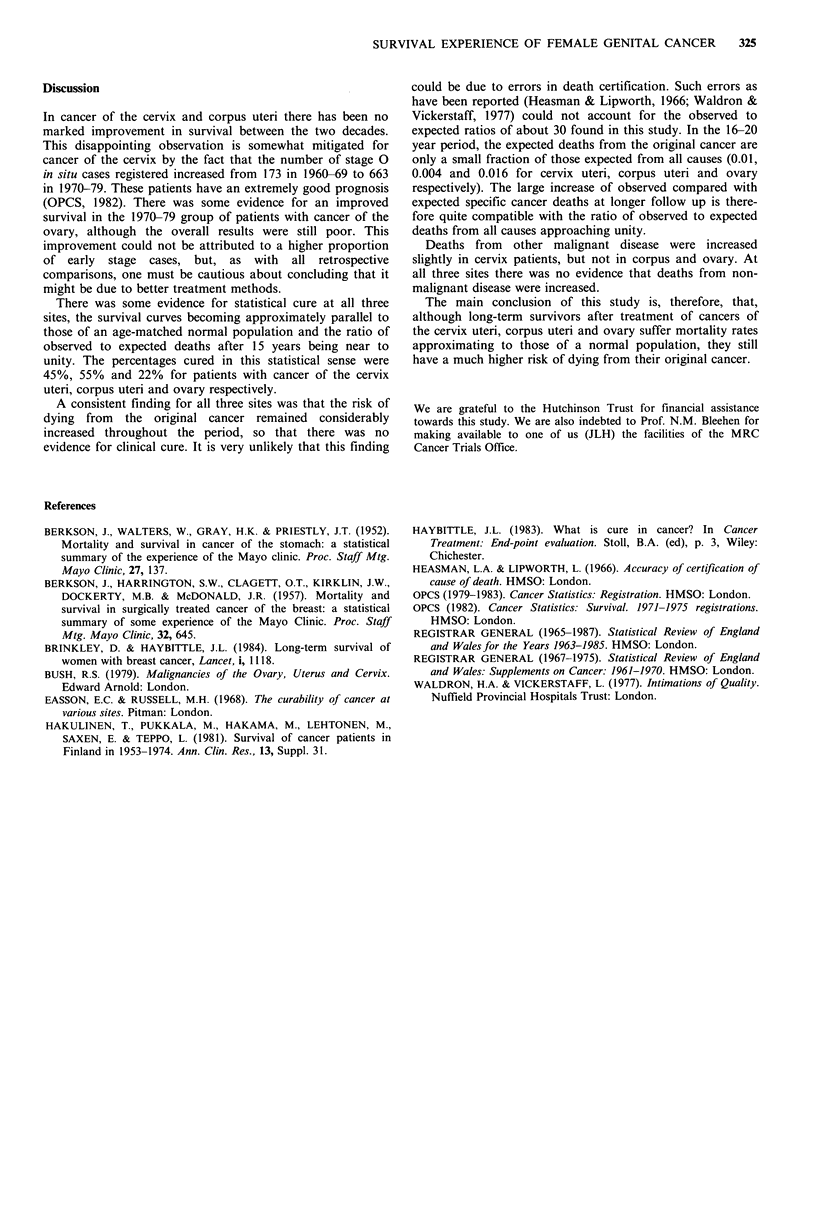# Long-term survival experience of female patients with genital cancer.

**DOI:** 10.1038/bjc.1988.73

**Published:** 1988-03

**Authors:** J. L. Haybittle, E. M. Kingsley-Pillers

**Affiliations:** MRC Cancer Trials Office, Cambridge, UK.

## Abstract

Survival analyses of patients with cancer of the cervix uteri, corpus uteri or ovary registered at Cambridge in 1960-1979 show that, although the long-term survivors had mortality rates similar to those of a normal age-matched population and might therefore be considered 'statistically cured', their risk of dying from their original cancer was still much higher than normal. Death rates from other cancers were slightly increased in cervix patients but not in corpus and ovary. At all three sites there was no evidence that deaths from non-malignant causes were increased. Only in cancer of the ovary was survival significantly better for patients registered in 1970-1979 than for patients registered in 1960-1969.


*Br. J. Cancer* (1988), **57**, 322–325 © The Macmillan Press Ltd., 1988

### Long-term survival experience of female patients with genital cancer

J.L. Haybittle[1] & E.M. Kingsley-Pillers[2]

[1]*MRC Cancer Trials Office, 5 Shaftesbury Road, Cambridge, CB2 2BW;* [2]*Cancer Registration Bureau, Addenbrooke's Hospital, Hills Road, Cambridge, CB2 2QQ, UK.*

**Summary** Survival analyses of patients with cancer of the cervix uteri, corpus uteri or ovary registered at Cambridge in 1960–1979 show that, although the long-term survivors had mortality rates similar to those of a normal age-matched population and might therefore be considered 'statistically cured', their risk of dying from their original cancer was still much higher than normal. Death rates from other cancers were slightly increased in cervix patients but not in corpus and ovary. At all three sites there was no evidence that deaths from non-malignant causes were increased. Only in cancer of the ovary was survival significantly better for patients registered in 1970–1979 than for patients registered in 1960–1969.

Several groups of female patients with genital cancer followed up beyond 10 years have been shown to have mortality rates approximating to those of a normal population of the same age distribution (Easson & Russell, 1968; Bush, 1979; Hakulinen *et al.*, 1981). This has usually been demonstrated by plotting the survival curve of the patients together with the expected survival curve for the similar normal population on a log-linear graph and observing parallelism between the curves at longer follow up. Berkson *et al.* (1952) first adopted this procedure for a group of stomach cancer patients, and in a subsequent similar study of breast cancer (Berkson *et al.*, 1957) they suggested that the long-term survivors, when the curves became parallel, were cured – 'at least statistically speaking'.

It has, however, been shown in patients with cancer of the breast that although the mortality rate from all causes after 25 years may not be very different from that of a normal population, the rate from breast cancer is still greatly increased, being about 15 times higher than expected (Brinkley & Haybittle, 1984). Thus, statistical cure is not necessarily the same as clinical cure, which has been defined as the complete elimination of the disease so that the patient would not have a higher risk of eventually dying from cancer at the original site than would persons of the same age and sex in the general population (Haybittle, 1983). We have, therefore, used the information available in the Cambridge Cancer Registration Bureau to see what evidence there is for clinical cure in patients suffering from cancer of either the cervix uteri, the corpus uteri or the ovary.

### Methods

All patients registered with cancer at these three sites from 1960 to 1979 inclusive were included in the study. They had a minimum follow up of 6 years and a maximum of 25 years. Expected deaths in a normal population were calculated using age-specific death rates for England and Wales adjusted by the East Anglian standardised mortality ratios (Registrar General, 1965–1987). Survival curves were obtained by the actuarial method with deaths grouped in yearly intervals. For the period beyond 5 years, the ratios of observed to expected deaths were calculated in successive 5-year periods to see if the ratios approached unity.

There had been continual development of treatment techniques over the study period. A subsidiary investigation was therefore made into any change in survival experience by dividing the patients into two groups, those registered in 1960–69 and those registered in 1970–79. The latter group had a maximum follow up of 16 years.

The results are presented in a similar format for each site separately and then followed by a general discussion.

### Cervix uteri

All patients except those in Stage O were included. There were 754 patients in 1960–69 and 788 in 1970–79.

*Comparison of the two periods* There was a significant difference ($P<0.001$) between the age distributions of the patients registered in the two periods (Figure 1). The distribution for the earlier period peaked at 40–44 years, while the maximum of the 1970–79 distribution was at 50–59 years. The latter group had a greater proportion of patients under 30 and a smaller proportion between 35 and 49 years old. This change is also shown in the overall figures for England and Wales (Registrar General, 1967–75; OPCS, 1979–83).

The survival curves for the two periods up to 16 years are shown in Figure 2a. Although the curve for 1970–79 lies above that for 1960–69, a comparison, unstandardised for

**Figure 1** Age distributions of patients registered with cancer of the cervix uteri in 1960–69 (-----) and 1970–79 (———).

Correspondence: J.L. Haybittle.
Received: 5 October, 1987; and in revised form 30 November, 1987.

**Figure 2** Survival curves for patients with cancer of (a) cervix uteri, (b) corpus uteri and (c) ovary registered in 1960–69 (-----) and 1970–79 (———).

stage, shows that the difference is not statistically significant ($0.3 > P > 0.2$). The proportion of stage I cases was in fact higher in the more recent period (Table I).

*Long-term survival* Figure 3a shows the survival curve for both groups combined and also the expected survival curve of a normal population of the same age distribution. Approximate parallelism is reached after 15 to 20 years. The lower dashed curve in Figure 3a, which is parallel to the normal population curve, is fitted by eye to the tail of the observed survival curve and indicates a statistically cured group of ~45%. The first row of Table II gives the ratios of observed to expected deaths from all causes together with the 95% confidence limits. Between 10 and 20 years the ratios are only just significantly greater than unity and a higher value than unity for 21–25 years cannot be excluded. The calculation for the last quinquennium is based on comparatively few patients, 109 entering the 21st year and only 26 entering the 25th year. However, after 10 years the death rate in the patients is not greatly increased over that in a normal population.

Nevertheless, the death rate from cancer of the cervix is still very much greater than expected (2nd row of Table II). No deaths from cancer of the cervix were recorded from 21 to 25 years but the wide confidence interval in this period shows that a large ratio of observed to expected deaths cannot be excluded.

Deaths from other malignant disease were increased slightly over the expected number (3rd row of Table II). Only in the 6–10 year period is the ratio of observed to expected significantly greater than unity, but, if the whole period 6–25 years is considered as one, the observed to expected ratio is 1.61 with 95% confidence limits of 1.15 and 2.19, i.e. again significantly greater than unity.

Deaths from non-malignant disease (4th row of Table II) were not significantly different from expected. The ratio for the whole 6–25 year period was 1.06 with 95% confidence limits of 0.82 and 1.35.

### *Corpus uteri*

The numbers of patients registered in 1960–69 and 1970–79 were 657 and 865 respectively.

**Table I** Stage distributions (%) in 1960–69 and 1970–79

| | | Stage | | | | |
|---|---|---|---|---|---|---|
| *Site* | *Period* | *I* | *II* | *III* | *IV* | *Unstaged* |
| Cervix uteri | 1960–69 | 38.4 | 33.9 | 18.8 | 7.6 | 1.4 |
| | 1970–79 | 45.3 | 30.5 | 16.3 | 5.8 | 2.0 |
| Corpus uteri | 1960–69 | 71.5 | 2.4 | 17.0 | 8.8 | 0.2 |
| | 1970–79 | 75.0 | 5.4 | 11.6 | 6.9 | 1.0 |
| Ovary | 1960–69 | 29.4 | 6.5 | 27.3 | 15.6 | 21.1 |
| | 1970–79 | 25.4 | 9.0 | 29.0 | 19.0 | 17.6 |

**Figure 3** Survival curves for patients registered in 1960–79 (———) with cancer of (a) cervix uteri, (b) corpus uteri and (c) ovary compared with curves for normal age-matched populations (-----). The lower dashed curves are parallel to the upper normal population curves and fitted by eye to the tails of the observed patient survival curves.

*Comparison of the two periods* The age distributions in the two periods were not significantly different ($0.1 > P > 0.05$). Both peaked at 55–64 years. The survival curves for the two periods (Figure 2b) are very similar although the percentage of patients in stages I and II were slightly higher in the later period (Table I).

*Long-term survival* Figure 3b shows the survival curve for the whole group of 1,522 patients together with the expected survival curve for a normal population of the same age distribution. Approximate parallelism occurs after about 12 years and, as indicated by the lower dashed curve, would suggest a statistically cured group of ~55%. The ratios of observed to expected deaths in successive quinquennia are given in Table III. For deaths from all causes, the ratio is just significantly greater than unity from 11 to 15 years, includes unity in its 95% confidence interval from 16 to 20 years, and is significantly less than unity from 21 to 25 years. Again, the calculation for the last quinquennium is based on comparatively small numbers; 111 entering the 21st year and 33 entering the 25th year.

The ratio of observed to expected deaths from cancer of the corpus uteri (2nd row of Table III) is much greater than unity in all the quinquennia except the last, and, as for cervix, a high ratio in 21–25 years cannot be excluded.

The 3rd row of Table III shows that there was no

**Table II** Ratio of observed (O) to expected (E) deaths with 95% confidence limits (CL) for patients with cancer of the cervix uteri

| | | *Period (years)* | | | | *Whole period* |
|---|---|---|---|---|---|---|
| Cause of death | | 6–10 | 11–15 | 16–20 | 21–25 | 6–25 |
| All causes | O/E | 2.93 | 1.46 | 1.54 | 0.54 | 2.03 |
| | CL | 2.40–3.54 | 1.05–1.98 | 1.01–2.24 | 0.11–1.59 | 1.74–2.35 |
| Ca. cervix | O/E | 110 | 39.4 | 27.8 | 0 | 69.0 |
| | CL | 83–144 | 21.0–67.3 | 9.0–65.0 | 0–83.6 | 53.9–86.9 |
| Other malignant | O/E | 1.81 | 1.65 | 1.42 | 0.74 | 1.61 |
| | CLk | 1.09–2.83 | 0.88–2.82 | 0.57–2.91 | 0.02–4.12 | 1.15–2.19 |
| Non-malignant | O/E | 1.33 | 0.75 | 1.20 | 0.49 | 1.06 |
| | CL | 0.92–1.86 | 0.42–1.24 | 0.67–1.98 | 0.06–1.76 | 0.82–1.35 |

**Table III** Ratio of observed (O) to expected (E) deaths with 95% confidence limits (CL) for patients with cancer of the corpus uteri

| | | *Period (years)* | | | | *Whole period* |
|---|---|---|---|---|---|---|
| Cause of death | | 6–10 | 11–15 | 16–20 | 21–25 | 6–25 |
| All causes | O/E | 1.92 | 1.34 | 1.09 | 0.41 | 1.45 |
| | CL | 1.63–2.26 | 1.05–1.68 | 0.78–1.48 | 0.16–0.84 | 1.28–1.63 |
| Ca. corpus | O/E | 121 | 56 | 31 | 0 | 79 |
| | CL | 92–157 | 32–91 | 10–72 | 0–60 | 63–96 |
| Other malignant | O/E | 0.90 | 1.26 | 1.00 | 1.11 | 1.04 |
| | CL | 0.53–1.44 | 0.70–2.07 | 0.40–2.06 | 0.23–3.24 | 0.75–1.41 |
| Non-malignant | O/E | 1.03 | 0.99 | 0.95 | 0.28 | 1.04 |
| | CL | 1.04–1.64 | 0.72–1.34 | 0.63–1.37 | 0.08–0.71 | 0.88–1.22 |

significant difference between observed and expected deaths from other malignant disease. The ratio of observed to expected for the whole period 6–25 years was 1.04 with 95% confidence limits of 0.75 and 1.41. Deaths from non-malignant disease (4th row of Table III) were similar to the expected numbers except in the last quinquennium where there is a suggestion that they might have been reduced. However, the ratio for the whole period 6–25 years was 1.04 with 95% confidence limits of 0.88 and 1.22.

*Ovary*

There were 1,474 patients in all, 617 in 1960–69 and 857 in 1970–79.

*Comparison of the two periods* There was no significant difference between the age distributions for the two periods ($0.20>P>0.10$). Their maxima were at 65–69 years.

The survival curves for the two periods (Figure 2c) were significantly different ($0.025>P>0.01$). At 15 years, the survival rates for the 1960–69 and 1970–79 patients were 14% and 19% respectively. An examination of the stage distribution in the two periods showed no improvement with time of the proportion of earlier stage cases, stages I and II accounting for about one third of the cases in both periods (Table I).

*Long-term survival* The survival curve for the whole group (Figure 3c) achieves approximate parallelism with that of the normal population after ~13 years and is consistent with there being a statistically cured group of 22%. The observed to expected ratios for deaths from all causes in the first row of Table IV are not significantly different from unity in the last two quinquennia. Because of the overall poor survival, 98 patients entered the 16th year and only 37 entered the 21st year.

Again there is no evidence for clinical cure. The death rate from cancer of the ovary was much higher than normal throughout the follow up (2nd row of Table IV).

Deaths from other malignant disease (3rd row of Table III) were not significantly different from expected. The observed to expected ratio for the whole period 6–25 years was 0.96 with 95% confidence limits of 0.48 and 1.73. Similarly for deaths from non-malignant disease (4th row of Table IV) where the ratio for the whole period was 0.79 with 95% confidence limits of 0.53 and 1.13.

**Table IV** Ratio of observed (O) to expected (E) deaths with 95% confidence limits (CL) for patients with cancer of the ovary

| | | *Period (years)* | | | | *Whole period* |
|---|---|---|---|---|---|---|
| Cause of death | | 6–10 | 11–15 | 16–20 | 21–25 | 6–25 |
| All causes | O/E | 2.94 | 1.90 | 1.18 | 0.56 | 2.21 |
| | CL | 2.31–3.70 | 1.27–2.75 | 0.54–2.25 | 0.07–2.03 | 1.78–2.66 |
| Ca. ovary | O/E | 129 | 38 | 25 | 28 | 84 |
| | CL | 98–167 | 17–71 | 5–73 | 0.7–155 | 66–106 |
| Other malignant | O/E | 0.85 | 1.53 | 0.60 | 0 | 0.96 |
| | CL | 0.28–2.0 | 0.50–3.6 | 0.02–3.3 | 0–5.9 | 0.48–1.73 |
| Non-malignant | O/E | 0.54 | 1.25 | 0.93 | 0.35 | 0.79 |
| | CL | 0.26–0.99 | 0.68–2.10 | 0.30–2.18 | 0.01–1.93 | 0.53–1.13 |

### Discussion

In cancer of the cervix and corpus uteri there has been no marked improvement in survival between the two decades. This disappointing observation is somewhat mitigated for cancer of the cervix by the fact that the number of stage O *in situ* cases registered increased from 173 in 1960–69 to 663 in 1970–79. These patients have an extremely good prognosis (OPCS, 1982). There was some evidence for an improved survival in the 1970–79 group of patients with cancer of the ovary, although the overall results were still poor. This improvement could not be attributed to a higher proportion of early stage cases, but, as with all retrospective comparisons, one must be cautious about concluding that it might be due to better treatment methods.

There was some evidence for statistical cure at all three sites, the survival curves becoming approximately parallel to those of an age-matched normal population and the ratio of observed to expected deaths after 15 years being near to unity. The percentages cured in this statistical sense were 45%, 55% and 22% for patients with cancer of the cervix uteri, corpus uteri and ovary respectively.

A consistent finding for all three sites was that the risk of dying from the original cancer remained considerably increased throughout the period, so that there was no evidence for clinical cure. It is very unlikely that this finding could be due to errors in death certification. Such errors as have been reported (Heasman & Lipworth, 1966; Waldron & Vickerstaff, 1977) could not account for the observed to expected ratios of about 30 found in this study. In the 16–20 year period, the expected deaths from the original cancer are only a small fraction of those expected from all causes (0.01, 0.004 and 0.016 for cervix uteri, corpus uteri and ovary respectively). The large increase of observed compared with expected specific cancer deaths at longer follow up is therefore quite compatible with the ratio of observed to expected deaths from all causes approaching unity.

Deaths from other malignant disease were increased slightly in cervix patients, but not in corpus and ovary. At all three sites there was no evidence that deaths from non-malignant disease were increased.

The main conclusion of this study is, therefore, that, although long-term survivors after treatment of cancers of the cervix uteri, corpus uteri and ovary suffer mortality rates approximating to those of a normal population, they still have a much higher risk of dying from their original cancer.

We are grateful to the Hutchinson Trust for financial assistance towards this study. We are also indebted to Prof. N.M. Bleehen for making available to one of us (JLH) the facilities of the MRC Cancer Trials Office.

### References

BERKSON, J., WALTERS, W., GRAY, H.K. & PRIESTLY, J.T. (1952). Mortality and survival in cancer of the stomach: a statistical summary of the experience of the Mayo clinic. *Proc. Staff Mtg. Mayo Clinic*, **27**, 137.

BERKSON, J., HARRINGTON, S.W., CLAGETT, O.T., KIRKLIN, J.W., DOCKERTY, M.B. & McDONALD, J.R. (1957). Mortality and survival in surgically treated cancer of the breast: a statistical summary of some experience of the Mayo Clinic. *Proc. Staff Mtg. Mayo Clinic*, **32**, 645.

BRINKLEY, D. & HAYBITTLE, J.L. (1984). Long-term survival of women with breast cancer, *Lancet*, **i**, 1118.

BUSH, R.S. (1979). *Malignancies of the Ovary, Uterus and Cervix*. Edward Arnold: London.

EASSON, E.C. & RUSSELL, M.H. (1968). *The curability of cancer at various sites*. Pitman: London.

HAKULINEN, T., PUKKALA, M., HAKAMA, M., LEHTONEN, M., SAXEN, E. & TEPPO, L. (1981). Survival of cancer patients in Finland in 1953-1974. *Ann. Clin. Res.*, **13**, Suppl. 31.

HAYBITTLE, J.L. (1983). What is cure in cancer? In *Cancer Treatment: End-point evaluation*. Stoll, B.A. (ed), p. 3, Wiley: Chichester.

HEASMAN, L.A. & LIPWORTH, L. (1966). *Accuracy of certification of cause of death*. HMSO: London.

OPCS (1979–1983). *Cancer Statistics: Registration*. HMSO: London.

OPCS (1982). *Cancer Statistics: Survival. 1971–1975 registrations*. HMSO: London.

REGISTRAR GENERAL (1965–1987). *Statistical Review of England and Wales for the Years 1963–1985*. HMSO: London.

REGISTRAR GENERAL (1967–1975). *Statistical Review of England and Wales: Supplements on Cancer: 1961–1970*. HMSO: London.

WALDRON, H.A. & VICKERSTAFF, L. (1977). *Intimations of Quality*. Nuffield Provincial Hospitals Trust: London.